# Participation of physical therapists in medical fee-based dialysis-prevention interventions: a nationwide survey in Japan

**DOI:** 10.1007/s10157-025-02763-z

**Published:** 2025-09-19

**Authors:** Yuma Hirano, Kenichi Kono, Ren Takahashi, Yuma Tamura, Momo Takahashi, Shinsuke Imaoka, Takuo Nomura, Makoto Igaki

**Affiliations:** 1 Department of Rehabilitation Medicine, Hamamatsu University Hospital, Hamamatsu City, Shizuoka Japan; 2 Department of Physical Therapy, International University of Health and Welfare School of Health Science at Fukuoka, 137-1 Enokizu, Okawa City, Fukuoka 831-8501 Japan; 3 Department of Rehabilitation, Kaikoukai Josai Hospital, Nagoya City, Aichi Japan; 4 Department of Rehabilitation, Nikko Medical Center, Dokkyo Medical University, Tochigi, Japan; 5 Department of Rehabilitation, Oita Oka Hospital, Oita City, Oita Japan; 6 Faculty of Rehabilitation, Kansai Medical University, Hirakata City, Osaka Japan; 7 Department of Rehabilitation, Toyooka Hospital, Toyooka Public Hospitals’ Association, Toyooka City, Hyogo Japan; 8 Working Group of Research and Development, Japanese Society of Physical Therapy for Diabetes Mellitus, Minato-Ku, Tokyo, Japan

**Keywords:** Physical therapists, Dialysis-prevention interventions, Medical fee, Healthcare system

## Abstract

**Background:**

Exercise is recommended to prevent dialysis; however, the involvement of physical therapists is not a criterion for reimbursable medical fee calculation in Japan. Consequently, eligible patients may not receive appropriate exercise guidance. We aimed to clarify the extent of physical therapist participation in dialysis-prevention interventions reimbursed under the current Japanese healthcare system and to identify reasons for non-participation related to reimbursement criteria.

**Methods:**

In January 2025, a 30-item questionnaire was distributed to all facility representatives registered with the Japan Physical Therapist Association to investigate medical fees and physical therapist involvement in dialysis prevention. Dialysis-prevention interventions were defined as those reimbursed under the Japanese healthcare system: Lifestyle-Related Disease Management, Diabetes Dialysis Prevention Guidance and Management (including Guidance of Patients with Severe Renal Impairment), and Chronic Kidney Disease (CKD) Dialysis Prevention Guidance and Management.

**Results:**

Of the 10,285 facilities surveyed, 1322 (12.9%) responded. Among these, physical therapists participated in Lifestyle-Related Disease Management, Diabetes Dialysis Prevention Guidance and Management, and CKD Dialysis Prevention Guidance and Management in 4.8%, 3.5%, and 2.3% of facilities, respectively. The most frequently cited reasons for exclusion were “Inclusion of physical therapists is not a strict requirement for medical fee reimbursement,” “Insufficient personnel or time,” and “No role assigned by the dialysis-prevention team.”

**Conclusion:**

Physical therapist involvement in dialysis-prevention interventions was limited, primarily due to current medical fee reimbursement criteria. Revising the healthcare system to facilitate their inclusion may enhance the delivery of exercise-based preventive care.

**Supplementary Information:**

The online version contains supplementary material available at 10.1007/s10157-025-02763-z.

## Introduction

Chronic kidney disease (CKD) is associated with increased risks of cardiovascular events, cardiovascular mortality, and all-cause death [1–3], while initiation of dialysis further reduces quality of life and increases healthcare costs [4]. In Japan, the government and academic societies aim to reduce annual new dialysis initiations to < 35,000 by 2028; however, the number reached 38,764 in 2023 [5], indicating that this target remains unmet. Current strategies for preventing dialysis initiation, therefore, require reassessment.

Dialysis-prevention interventions encompass dietary, lifestyle, and exercise counseling [6], with exercise shown to slow renal function decline. In Japan, exercise guidance is included in reimbursable services such as Lifestyle-Related Disease Management, Diabetes Dialysis Prevention Guidance and Management (including Guidance of Patients with Severe Renal Impairment), and CKD Dialysis Prevention Guidance and Management. These align with the consensus that, in everyday clinical practice, renal rehabilitation should be delivered by a multidisciplinary team, comprising nephrologists, nurses, dietitians, and exercise professionals, and ideally reimbursed by the national healthcare system [7]. However, reimbursement criteria do not mandate the inclusion of physical therapists or exercise specialists. A study examining multidisciplinary care teams found that including physical therapists significantly reduced the hazard ratio for a composite endpoint of renal replacement therapy initiation and mortality [8]. Their absence in dialysis-prevention interventions may result in inadequate exercise guidance and insufficient attenuation of renal function decline.

However, the extent of physical therapist involvement in dialysis prevention and the reason for their non-participation remain unclear. This study primarily aimed to determine the proportion of physical therapists participating in reimbursement-based dialysis-prevention interventions, and secondarily to identify reasons for their exclusion.

## Materials and methods

### Study design and population

This nationwide, cross-sectional online survey was conducted in Japan by the Working Group of the Research Promotion Committee of the Japanese Society of Physical Therapy for Diabetes Mellitus between January 6 and January 31, 2025. Invitations were sent via institutional email to representatives of all 10,285 medical facilities registered with the Japanese Physical Therapy Association, requesting completion of a 30-item online questionnaire. Under Japan’s healthcare system, medical costs for dialysis-prevention interventions, including Lifestyle-Related Disease Management, Diabetes Dialysis Prevention Guidance and Management (which includes Guidance of Patients with Severe Renal Impairment), and CKD Dialysis Prevention Guidance and Management, are reimbursable if facilities meet detailed requirements, including specific facility standards. Adoption of these services depends on implementation costs and operational feasibility. The questionnaire comprised six items on basic facility information, seven on Lifestyle-Related Disease Management, nine on Diabetes Dialysis Prevention Guidance and Management (including the Guidance of Patients with Severe Renal Impairment), and eight on the CKD Dialysis Prevention Guidance and Management. The questionnaire primarily asked whether each reimbursable service was billed at the respondent’s institution, the proportion of cases involving physical therapist participation, and reasons for non-participation (Online Resource 1). It was developed by Working Group members, led by Y.T. and M.T., based on literature review [9, 10] and clinical experience, and was independently reviewed for content validity by Research Promotion Committee of the Japanese Society of Physical Therapy for Diabetes Mellitus not involved in its development. Invitations were sent to institutional email addresses; the survey required approximately 10 min to complete. No follow-up reminder emails were issued, and the invitation was distributed only once, on the first day of the survey period. Responses were submitted online, and no monetary incentives were provided to respondents.

### Definition of dialysis-prevention interventions and overview of reimbursement

Interventions included in the reimbursement schedule—Lifestyle-Related Disease Management, Diabetes Dialysis Prevention Guidance and Management (including the Guidance of Patients with Severe Renal Impairment), and CKD Dialysis Prevention Guidance and Management—were classified as dialysis-prevention interventions. A summary of the reimbursement details for each patient is provided in Table 1.

**Table 1:** Overview of medical fees

	Lifestyle-related disease management fee	Diabetes dialysis prevention guidance and management fee	Fee for guidance of patients with severe renal impairment	CKD dialysis prevention guidance and management fee
Points	570–720 points (once monthly)	350 points (once monthly)	100 points	250–300 points (once monthly)
Eligible patients	Patients with dyslipidemia, hypertension, or diabetes	Patients with HbA1c ≥ 6.1% (JDS) or receiving oral hypoglycemic agents/insulin, with diabetic nephropathy ≥ Stage 2	Of those billed under the diabetes dialysis prevention guidance and management fee, patients with eGFR < 45 mL/min/1.73 m^2^	Non-hospitalized patients with chronic kidney disease who are classified as high risk for dialysis according to CKD severity staging
Overview	Billable when comprehensive lifestyle-related management (medication adherence, exercise, nutrition, etc.) is conducted according to a treatment plan	Billable when the “Dialysis Prevention Clinical Team” delivers individualized dietary guidance, exercise instruction, and other lifestyle counseling	Billable when a physician provides instruction on exercise type, frequency, intensity, duration, and precautions to maintain renal function	Billable when the “Dialysis Prevention Clinical Team,” based on the Japanese Society of Nephrology’s Evidence-based CKD Practice Guidelines, provides individualized interventions as needed, including disease-stage classification, dietary guidance on salt and protein restriction, exercise instruction, and other lifestyle counseling
Team composition	Comprehensive treatment management may be carried out in collaboration with nurses, pharmacists, registered dietitians, and other allied professionals	Must include a physician, a nurse or public health nurse, and a registered dietitian	Physician	“The Dialysis Prevention Clinical Team"must include a physician, a nurse or public health nurse, and a registered dietitian

Fees in Japan’s national schedule are expressed as points; 1 point = JPY 10. The totals shown are pre-cost-sharing amounts. Patients pay a co-payment based on their statutory coinsurance rate (e.g., 30% for most adults). For example, 660 points = JPY 6600 in total; with 30% coinsurance, the patient pays JPY 1980

Adoption of reimbursement schemes is decided at the institutional level after reviewing staffing, expected patient volume, documentation requirements, and claim-denial risk. Each scheme specifies the minimum team composition required for billing (e.g., physician, nurse, dietitian, pharmacist). Physical therapists are not mandated; facilities may additionally involve physical therapists under physician supervision, with their contribution bundled within the scheme and not reimbursed separately

*JDS* Japanese Diabetes Society, *JSN* Japanese Society of Nephrology, *CKD* chronic kidney disease, *eGFR* estimated glomerular filtration rate

### Statistical analysis

Facility characteristics are summarized as counts and percentages. For each reimbursable medical fee, characteristics of facilities with versus without physical therapist participation were compared using the Chi-squared (*χ*^2^) test or Fisher’s exact test, as appropriate. All statistical analyses were performed using R software (version 4.4.1; The R Foundation, Vienna, Austria), with a two-tailed significance level set at 5%.

## Results

### Survey response rate and facility characteristics

Of the 10,285 facilities surveyed, 1322 responded, yielding a 12.9% response rate. Approximately 50% of the responding facilities were hospitals (medical corporations), 36.4% had 100–299 beds, and 3.9% had ≥ 600. Furthermore, approximately 70–80% had both internal medicine and rehabilitation departments, and 47.1% employed 1–9 physical therapists. Among the patient populations targeted by each facility, outpatients were the most common (84.0%), followed by acute-phase patients (52.6%) (Table 2).

**Table 2:** Characteristics of the subject facility

	All participants *n* = 1322
Working facility	
Hospital (affiliated with university)	57 (4.3)
Hospital (national government, public medical institutions, social insurance-related organizations)	229 (17.3)
Hospital (medical corporation)	622 (47.0)
Hospital (private practice)	37 (2.8)
Clinic (medical corporation)	228 (17.2)
Clinic (private practice)	110 (8.3)
Others	39 (3.0)
Number of hospital beds	
600 or more beds	52(3.9)
300–599 beds	184 (13.9)
100–299 beds	481 (36.4)
1–99 beds	309 (23.4)
Without a bed	296 (22.4)
Clinical specialties (departments)	
Internal medicine	1007 (76.2)
Cardiology	624 (47.2)
Nephrology	353 (26.7)
Diabetes/metabolism	429 (32.5)
Dialysis departments	430 (32.5)
Urology	493 (37.3)
Rehabilitation	1113 (84.2)
Orthopedics departments	977 (73.9)
Not applicable	22 (1.7)
Facility standards for disease-specific rehabilitation	
Cardiac Rehabilitation Fee (I)	358 (27.1)
Cardiac Rehabilitation Fee (II)	17 (1.3)
Cerebrovascular Disorders Rehabilitation Fee (I)	601 (45.5)
Cerebrovascular Disorders Rehabilitation Fee (II)	245 (18.5)
Cerebrovascular Disorders Rehabilitation Fee (III)	168 (12.7)
Disuse Syndrome Rehabilitation Fee (I)	597 (45.2)
Disuse Syndrome Rehabilitation Fee (II)	182 (13.8)
Disuse Syndrome Rehabilitation Fee (III)	97 (7.3)
Musculoskeletal Rehabilitation Fee (I)	978 (74.0)
Musculoskeletal Rehabilitation Fee (II)	192 (14.5)
Musculoskeletal Rehabilitation Fee (III)	83 (6.3)
Respiratory Rehabilitation Fee (I)	683 (51.7)
Respiratory Rehabilitation Fee (II)	63 (4.8)
Not applicable	61 (4.6)
Number of physical therapists	
30 or more	229 (17.3)
20–29	171 (12.9)
10–19	299 (22.6)
1–9	623 (47.1)
Disease phase of the patients covered	
Intensive care unit	242 (18.3)
Acute phase	695 (52.6)
Convalescent rehabilitation phase	397 (30.0)
Community-based integrated medical care	132 (10.0)
Community-based care	424 (32.1)
Outpatients phase	1110 (84.0)
Home visits	535 (40.5)
Day-service programs	367 (27.8)
Not applicable	44 (3.3)

Values are shown as *n* (%)

In Japan’s fee schedule, claims for disease-specific rehabilitation (e.g., cardiac, cerebrovascular, disuse syndrome, musculoskeletal, respiratory) are allowed only at facilities certified as meeting government-defined *facility standards*. These standards generally require: a designated rehabilitation service under physician supervision; licensed PT/OT/ST staffing; availability of therapy on specified days/hours; individualized plans and documentation; regular multidisciplinary case conferences; appropriate equipment/safety systems; and basic quality management. Facilities not certified cannot bill these items even if similar services are provided

*CKD* chronic kidney disease

### Ratio of facilities providing the reimbursable services

**Fig. 1:**
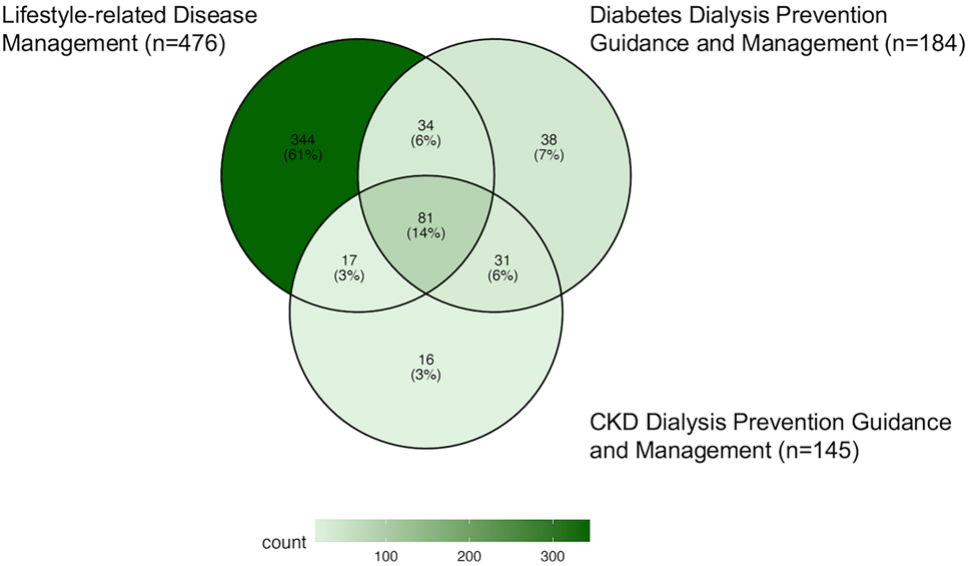
Proportion of facilities billing multiple interventions. Among all facilities billing the Lifestyle‐related Disease Management (*n* = 476), the Diabetes Dialysis Prevention Guidance and Management (*n* = 184), and the CKD Dialysis Prevention Guidance and Management (*n* = 145), this figure shows the proportion of facilities that claim more than one of these interventions. The Venn diagram visually represents the overlap in billing among facilities claiming these three interventions, illustrating the percentages that bill each intervention alone as well as in combination

Overall, 1322 facilities responded, of which 476 (36.0%) billed the Lifestyle-Related Disease Management Fee, 184 (13.9%) billed the Diabetes Dialysis Prevention Guidance and Management Fee, and 145 (11.0%) billed the CKD Dialysis Prevention Guidance and Management Fee. Among these, only 81 (14.4%) billed for all three interventions (Fig. 1). Among the facilities not currently billing these medical fees, 59 (4.5%), 73 (5.5%), and 67 (5.1%) were in the process of arranging to bill the Lifestyle-Related Disease Management Fee, Diabetes Dialysis Prevention Guidance and Management Fee, and CKD Dialysis Prevention Guidance and Management Fee, respectively. The most frequently cited reason for not billing these medical fees was failure to meet facility criteria (Table 3).

**Table 3:** Reported reasons for not claiming medical fee-based intervention

	Lifestyle-related disease management (*n* = 846)	Diabetes dialysis prevention guidance and management (*n* = 1138)	CKD dialysis prevention guidance and management (*n* = 1177)
Failure to meet the facility criteria	322 (38.1)	467 (41.0)	486 (41.3)
No relevant department or specialist physicians available	272 (32.2)	398 (35.0)	421 (35.8)
Unable to form the dialysis‐prevention clinical team	–	348 (30.6)	363 (30.8)
Insufficient non-physician staff and inability to coordinate multidisciplinary collaboration	207 (24.5)	–	–
Dialysis‐prevention team members have not completed the required training for preventive guidance	–	–	277 (23.5)
No eligible patients	139 (16.4)	218 (19.2)	229 (19.5)
Unaware of the existence of each medical fee	172 (20.3)	168 (14.8)	172 (14.6)
Do not perceive a need to bill for it as a medical institution		79 (6.9)	78 (6.6)
Other	19 (2.2)	19 (1.7)	21 (1.8)
Unknown	123 (14.5)	198 (17.4)	210 (17.8)

For each intervention, reasons for non-claiming were tallied in a multiple‐response format. For each intervention, the denominator was the number of facilities not billing that fee, and the number (%) of facilities citing each reason is presented

*CKD* chronic kidney disease

### Physical therapists’ participation rates in dialysis-prevention interventions

**Fig. 2:**
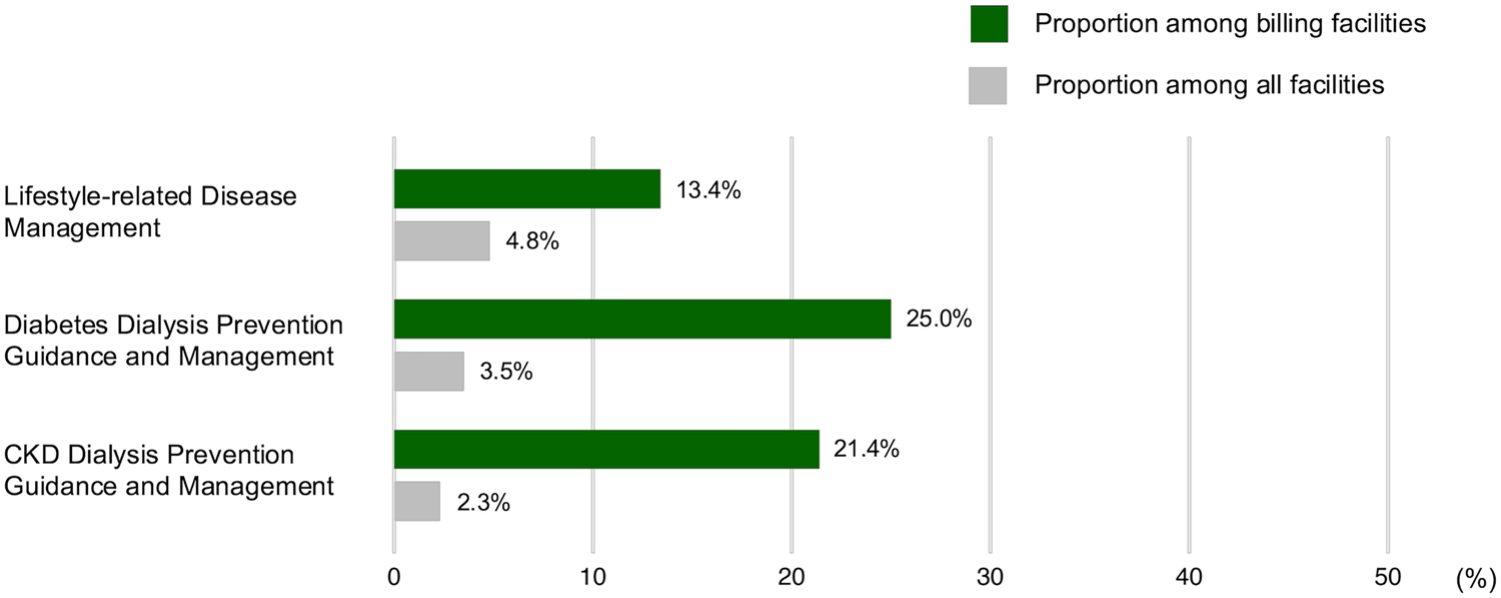
Physical therapist participation rates in dialysis-prevention interventions-related medical fee. The bar graph shows the proportion of facilities with physical therapist involvement in interventions billed under the Lifestyle-related Disease Management, the Diabetes Dialysis Prevention Guidance and Management, and the CKD Dialysis Prevention Guidance and Management. Dark green bars represent the percentage among facilities billing each intervention, and gray bars represent the percentage among all surveyed facilities

Physical therapists participated in the Lifestyle-Related Disease Management intervention at 64 facilities, corresponding to 13.4% of billing facilities and 4.8% of all respondents (Fig. 2). They participated in the Diabetes Dialysis Prevention Guidance and Management at 46 facilities, corresponding to 25.0% of the billing facilities and 3.5% of all respondents. Among the facilities that billed the Diabetes Dialysis Prevention Guidance and Management Fee, physical therapists participated in the Guidance of Patients with Severe Renal Impairment at four facilities: one billed 10–14 patients per month, one billed 5–9, and the remaining two billed < 5. Physical therapists participated in the CKD Dialysis Prevention Guidance and Management at 31 facilities, representing 21.4% of the billing facilities, and 2.3% of all respondents. In these participating facilities, the monthly number of patients billed was ≥ 20 in 3 facilities (9.7%), 10–14 in 4 (12.9%), 5–9 in 10 (32.3%), and < 5 in 14 (45.2%).

### Reasons for physical therapists’ non-participation in reimbursement-based dialysis-prevention interventions

Facilities including physical therapists’ services in the Lifestyle-Related Disease Management Fee had a significantly higher percentage of cardiology (*p* = 0.03), nephrology (*p* < 0.01), diabetes/metabolism (*p* = 0.01), and dialysis departments (*p* = 0.02), and a significantly lower percentage of orthopedic departments (*p* = 0.05) compared with facilities without such inclusion. The proportion of facilities targeting intensive care unit patients was also significantly higher (*p* < 0.01). Facility characteristics did not differ significantly between those with and without the inclusion of physiotherapy services in the Diabetes Dialysis Prevention Guidance and Management Fee. Facilities including physical therapists’ services in the CKD Dialysis Prevention Guidance and Management Fee had a significantly lower proportion of urology departments compared with those without inclusion (*p* < 0.01) (Table 4).

**Table 4:** Comparison of facility characteristics

	Surveyed facilities (*n* = 1322)								
	Billing for the lifestyle-related disease management fee *n* = 476 (36.0)			Billing for the diabetes dialysis prevention guidance and management Fee *n* = 184 (13.9)			Billing for the CKD dialysis prevention guidance and management fee *n* = 145 (11.0)		
	PT Participate *n* = 64	PT Not participate *n* = 412	*p*	PT Participate *n* = 46	PT Not participate *n* = 138	*p*	PT Participate *n* = 31	PT Not participate *n* = 114	*p*
Working facility									
Hospital (affiliated with university)	1 (1.6)	8 (1.9)	0.93	4 (8.7)	13 (9.4)	0.60	1 (3.2)	10 (8.8)	0.65
Hospital (national government, public medical institutions, social insurance related organizations)	10 (15.6)	61 (14.8)		13 (28.3)	46 (33.3)		8 (25.8)	39 (34.2)	
Hospital (medical corporation)	31 (48.4)	218 (52.9)		26 (56.5)	62 (44.9)		18 (58.1)	50 (43.9)	
Hospital (private practice)	2 (3.1)	8 (1.9)		1 (2.2)	1 (0.7)		1 (3.2)	1 (0.9)	
Clinic (medical corporation)	15 (23.4)	79 (19.2)		2 (4.3)	7 (5.1)		2 (6.5)	9 (7.9)	
Clinic (private practice)	4 (6.2)	32 (7.8)		0 (0.0)	3 (2.2)		0 (0.0)	1 (0.9)	
Others	1 (1.6)	6 (1.5)		0 (0.0)	6 (4.3)		1 (3.2)	4 (3.5)	
Number of hospital beds									
600 or more beds	0 (0.0)	10 (2.4)	0.09	4 (8.7)	18 (13.0)	0.40	3 (9.7)	14 (12.3)	0.90
300〜599 beds	9 (14.1)	22 (5.3)		8 (17.4)	37 (26.8)		7 (22.6)	33 (28.9)	
100〜299 beds	25 (39.1)	153 (37.1)		24 (52.2)	52 (37.7)		12 (38.7)	41 (36.0)	
1〜99 beds	20 (31.2)	134 (32.5)		10 (21.7)	28 (20.3)		8 (25.8)	23 (20.2)	
Without a bed	10 (15.6)	93 (22.6)		0 (0.0)	3 (2.2)		1 (3.2)	3 (2.6)	
Clinical specialties (departments)									
Internal medicine	58 (90.6)	373 (90.5)	1.00	44 (95.7)	126 (91.3)	0.52	29 (93.5)	111 (97.4)	0.29
Cardiology	43 (67.2)	214 (51.9)	0.03^*^	40 (87.0)	108 (78.3)	0.28	28 (90.3)	89 (78.1)	0.20
Nephrology	29 (45.3)	111 (26.9)	< 0.01^**^	33 (71.7)	91 (65.9)	0.59	23 (74.2)	87 (76.3)	0.99
Diabetes/metabolism	33 (51.6)	143 (34.7)	0.01^*^	35 (76.1)	94 (68.1)	0.40	22 (71.0)	79 (69.3)	1.00
Dialysis departments	32 (50.0)	139 (33.7)	0.02^*^	38 (82.6)	98 (71.0)	0.17	27 (87.1)	96 (84.2)	0.79
Urology	22 (34.4)	147 (35.7)	0.95	29 (63.0)	99 (71.7)	0.35	16 (51.6)	89 (78.1)	< 0.01^**^
Rehabilitation	55 (85.9)	353 (85.7)	1.00	43 (93.5)	126 (91.3)	0.76	28 (90.3)	107 (93.9)	0.45
Orthopedics departments	40 (62.5)	310 (75.2)	0.05^*^	41 (89.1)	117 (84.8)	0.63	24 (77.4)	92 (80.7)	0.88
Not applicable	0 (0)	1 (0.2)	1.00	0 (0.0)	0 (0.0)	–	0 (0.0)	0 (0.0)	–
Number of physical therapists									
30 or more	12 (18.8)	58 (14.1)	0.66	13 (28.3)	32 (23.2)	0.89	9 (29.0)	21 (18.4)	0.39
20–29	8 (12.5)	46 (11.2)		8 (17.4)	28 (20.3)		5 (16.1)	24 (21.1)	
10–19	17 (26.6)	103 (25.0)		14 (30.4)	46 (33.3)		8 (25.8)	43 (37.7)	
1–9	27 (42.2)	205 (49.8)		11 (23.9)	32 (23.2)		9 (29.0)	26 (22.8)	
Disease phase of the patients covered									
Intensive Care Unit	15 (23.4)	38 (9.2)	< 0.01^**^	17 (37.0)	57 (41.3)	0.73	12 (38.7)	45 (39.5)	1.00
Acute phase	39 (60.9)	217 (52.7)	0.27	42 (91.3)	117 (84.8)	0.38	25 (80.6)	95 (83.3)	0.93
Convalescent rehabilitation phase	22 (34.4)	118 (28.6)	0.43	17 (37.0)	39 (28.3)	0.35	10 (32.3)	35 (30.7)	1.00
Community-based integrated medical care	8 (12.5)	52 (12.6)	1.00	7 (15.2)	20 (14.5)	1.00	2 (6.5)	19 (16.7)	0.25
Community-based care	22 (34.4)	177 (43.0)	0.25	23 (50.0)	56 (40.6)	0.34	11 (35.5)	49 (43.0)	0.59
Outpatients phase	60 (93.8)	358 (86.9)	0.18	44 (95.7)	122 (88.4)	0.25	27 (87.1)	101 (88.6)	0.76
Home visits	29 (45.3)	224 (54.4)	0.22	15 (32.6)	50 (36.2)	0.79	9 (29.0)	40 (35.1)	0.68
Day-service programs	22 (34.4)	152 (36.9)	0.80	12 (26.1)	29 (21.0)	0.61	6 (19.4)	29 (25.4)	0.64
Not applicable	0 (0.0)	9 (2.2)	0.62	0 (0.0)	2 (1.4)	1.00	0 (0.0)	0 (0.0)	–

Among facilities claiming medical fee-based intervention, the number and proportion of facilities with and without physical therapist involvement are presented by facility characteristics

Clinical specialties and patient disease phases were recorded using a multiple-response format

*PT* physical therapist, *CKD* chronic kidney disease

^*^ *p* < 0.05, ** *p* < 0.01

**Fig. 3:**
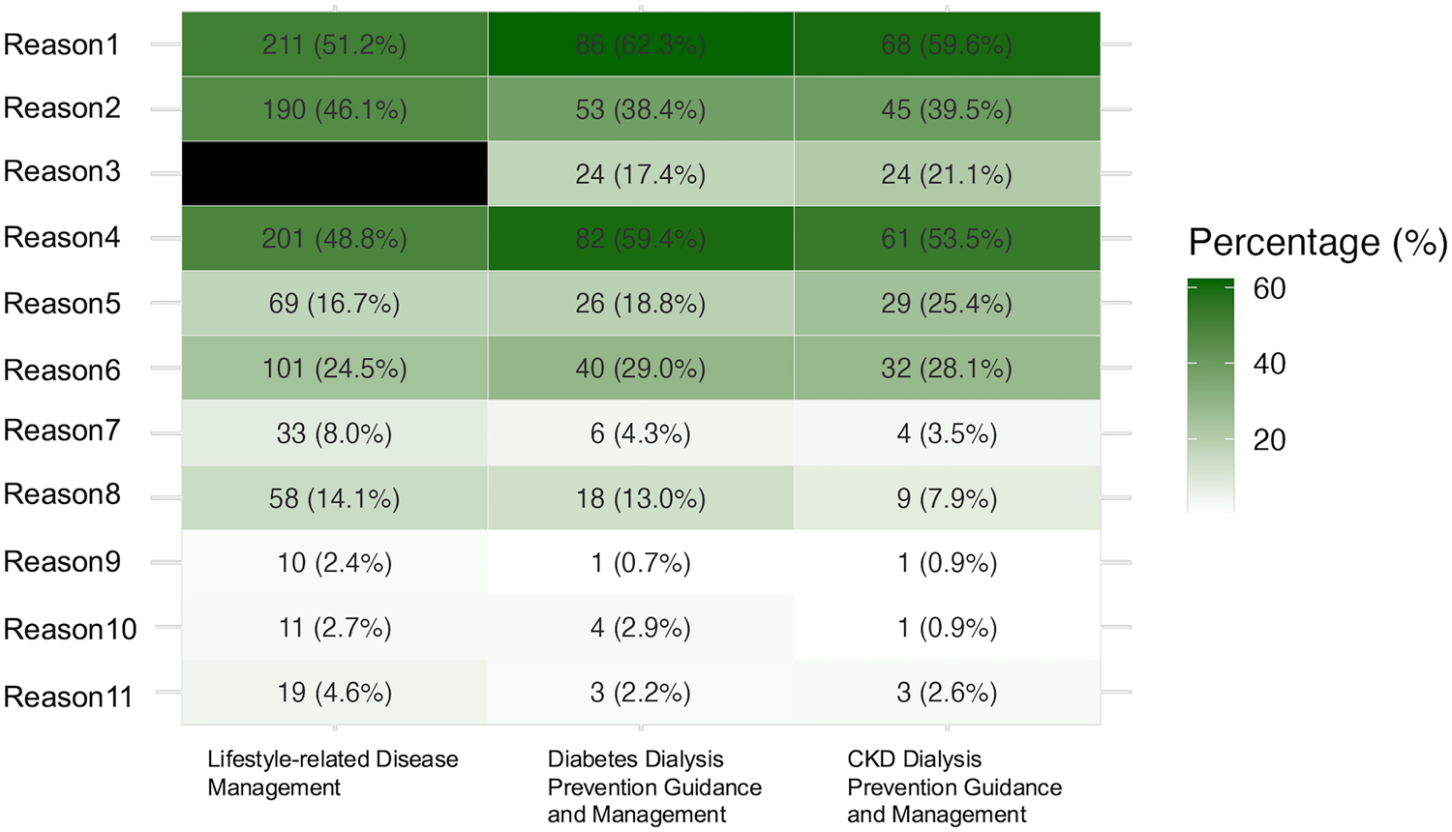
Reasons for the absence of physical therapist participation in dialysis‐prevention interventions billed under each intervention. A heat map illustrates the reasons why physical therapists do not participate, despite the facility billing the Lifestyle‐related Disease Management, the Diabetes Dialysis Prevention Guidance and Management, or the CKD Dialysis Prevention Guidance and Management. The color intensity represents the percentage of respondents selecting each reason, with darker shades indicating more frequently chosen reasons. Reasons 1–11 are defined as follows: Reason1: The assignment of physiotherapists is not a requirement for meeting the reimbursement criteria. Reason2: They were not assigned a role by the dialysis-prevention team. Reason3: It is difficult to provide instruction on the same day as other healthcare professionals. Reason4: There is insufficient personnel or time available. Reason5: Low cost‐effectiveness. Reason6: It does not directly generate revenue for the rehabilitation department. Reason7: Lack of collaboration with the department physicians (internal medicine specialists). Reason8: No physical therapists with the necessary specialized knowledge and skills are available. Reason9: Lack of perceived interest or necessity within the physical therapy department. Reason10: Others. Reason11: Unknown

Across all three medical fees, most common reasons for excluding physical therapists’ services from medical fee billing were: “The assignment of physical therapists is not a requirement for meeting the reimbursement criteria” (51.2%, 62.3%, and 59.6%, respectively), “Insufficient personnel or time availability” (48.8%, 59.4%, and 53.5%, respectively), and “No role assigned by the dialysis- prevention team” (46.1%, 38.4%, and 39.5%, respectively). The least common reason was “Lack of perceived interest or necessity within the physical therapy department” (2.4%, 0.7%, and 0.9%, respectively) (Fig. 3).

### Physical therapist involvement in dialysis-prevention interventions

In facilities that included physical therapists’ services in the Lifestyle-Related Disease Management, Diabetes Dialysis Prevention Guidance and Management, and CKD Dialysis Prevention Guidance and Management Fees, 70.3%, 65.2%, and 74.2%, respectively, reported “providing direct exercise instruction and related interventions to patients under physician orders,” while 23.4%, 54.3%, and 38.7%, respectively, reported that “only a subset of highly specialized physical therapists are involved” (Table 5). The most frequently reported forms of exercise guidance were aerobic exercise and exercise prescription (76.6%, 76.1%, and 80.6%, respectively), resistance exercise and exercise prescription (65.6%, 67.4%, and 74.2%), guidance on increasing physical activity (62.5%, 67.4%, and 64.5%), and stretching exercises (59.4%, 69.6%, and 67.7%). Contrastingly, motivational counseling based on the stages of behavior change was the least frequently reported (25.0%, 34.8%, and 19.4%) (Table 6). Furthermore, approximately half the facilities reported engaging with fewer than 25% of all patients (56.3%, 52.3%, and 45.2%, respectively) (Table 7).

**Table 5:** Methods of physical therapist involvement in billing for each intervention

	Lifestyle-related disease management (*n* = 64)	Diabetes dialysis prevention guidance and management (*n* = 46)	CKD dialysis prevention guidance and management (*n* = 31)
Providing direct exercise instruction and related interventions to patients under physician orders	46 (70.3)	30 (65.2)	23 (74.2)
Developing exercise‐instruction materials used by other professionals	17 (26.6)	16 (34.8)	14 (45.2)
Training other professionals in how to deliver exercise guidance	10 (15.6)	10 (21.7)	8 (25.8)
Working in the rehabilitation room	21 (32.8)	17 (37.0)	7 (22.6)
Working outside the rehabilitation room (e.g., in the consultation room or on the ward)	18 (28.1)	15 (32.6)	6 (19.4)
Participating concurrently with disease‐specific rehabilitation sessions	17 (26.6)	13 (28.3)	9 (29.0)
Participating outside of disease‐specific rehabilitation hours (e.g., at lunchtime or after 5 pm)	4 (6.2)	2 (4.3)	1 (3.2)
Only a subset of highly specialized physical therapists are involved	15 (23.4)	25 (54.3)	12 (38.7)
Many physical therapists are involved regardless of specialization	9 (14.1)	2 (4.3)	2 (6.5)
Other (please describe your approach in detail)	5 (7.8) ^a^	4 (8.7) ^b^	0 (0.0)

Methods of physical therapist involvement for each intervention were tabulated in a multiple‐response format. For each intervention, the denominator was the number of facilities with physical therapist participation, and the number (%) of facilities selecting each involvement method is presented

*CKD* chronic kidney disease

^a^Participated in team management; delivered lectures in diabetes education classes; provided aquatic exercise therapy in the pool; conducted interviews and evaluations based on individualized goal setting

^b^Offered advice during conferences; managed exercise pamphlets; participated in committee activities; engaged in team management

**Table 6:** Exercise guidance content in billing for each intervention

	Lifestyle‐related disease management (*n* = 64)	Diabetes dialysis prevention guidance and management (*n* = 46)	CKD dialysis prevention guidance and management (*n* = 31)
Aerobic exercise and exercise prescription	49 (76.6)	35 (76.1)	25 (80.6)
Resistance exercise and exercise prescription	42 (65.6)	31 (67.4)	23 (74.2)
Guidance on increasing physical activity (including the use of activity monitors such as accelerometers and pedometers)	40 (62.5)	31 (67.4)	20 (64.5)
Stretching exercises	38 (59.4)	32 (69.6)	21 (67.7)
Motivational counseling based on the stages of behavior change	16 (25.0)	16 (34.8)	6 (19.4)
Others	5 (7.8)^a^	7 (15.2)^b^	3 (9.7)^c^

Types of exercise guidance were tallied in a multiple‐response format. For each intervention, facilities with physical therapist participation in billing that intervention served as the denominator, and the number (%) of facilities providing each guidance type are shown

*CKD* chronic kidney disease

^a^Providing movement guidance; communicating care plans to community service coordinators; adjusting and instructing on exercise intensity/load; foot care

^b^Conducting regular assessments; hosting kidney‐disease education classes; distributing educational materials; building multidisciplinary support systems; foot care

^c^Conducting regular assessments; foot care

**Table 7:** Proportion of patients receiving physical therapist intervention in facilities with physical therapist involvement in billing each intervention

	Lifestyle‐related disease management (*n* = 64)	Diabetes dialysis prevention guidance and management (*n* = 46)	CKD dialysis prevention guidance and management (*n* = 31)
≥ 75%	8 (12.5)	7 (15.2)	5 (16.1)
≥ 50% but < 75%	7 (10.9)	5 (10.9)	1 (3.2)
≥ 25% but < 50%	13 (20.3)	8 (17.4)	11 (35.5)
< 25%	36 (56.3)	24 (52.3)	14 (45.2)
Only when billing the “Guidance of Patients with Severe Renal Impairment (eGFR < 45 mL/min/1.73 m^2^)”	–	2 (4.3)	–

Among facilities with physical therapist participation in billing each intervention, the percentage of all patients billed under that intervention who receive physical therapist involvement is shown

*CKD* chronic kidney disease

## Discussion

In our survey of medical facilities employing physical therapists, we assessed the proportion of physical therapists participating in dialysis-prevention interventions reimbursable under the current medical fee schedule and explored reasons for non-participation. Among responding facilities, 4.8% of physical therapists were involved in Lifestyle-Related Disease Management, 3.5% in Diabetes Dialysis Prevention Guidance and Management, and 2.3% in CKD Dialysis Prevention Guidance and Management, indicating low participation in these reimbursable interventions. Commonly cited barriers to participation included: “The assignment of physical therapists is not a requirement for meeting reimbursement criteria,” “Insufficient personnel or time availability,” and “No role assigned by the dialysis-prevention team.” These findings underscore the need to incorporate physical therapist staffing into reimbursement requirements to facilitate their involvement in dialysis-prevention interventions.

Accumulating evidence suggests that exercise and physical activity attenuate the decline in renal function and delay the initiation of dialysis [6]. Although healthcare professionals acknowledge this importance [11, 12], only 10–20% of facilities worldwide offer such interventions to patients with CKD, indicating a global scarcity [11, 13, 14]. An international survey revealed a substantial shortage of exercise programs for patients with CKD [12]. Reported barriers include limited staff motivation and interest, insufficient confidence or time among specialists to provide counseling, and a shortage of trained personnel such as physical therapists and other exercise professionals [14–16].

The involvement of physical therapists in caring for patients with CKD is notably low. A 2017 survey reported that only 4.5% participated in medical fee-based dialysis-prevention interventions [17]. In the USA, only 2.2% are involved in outpatient diabetes care [18]; in the UK, only 7% of facilities provide rehabilitation for conservative CKD management [19]. These findings align with earlier studies, highlighting the insufficient engagement of physical therapists in dialysis prevention.

Patients with type 2 diabetes have expressed a preference for the inclusion of physical therapists or other exercise-promotion specialists in their care team, noting that guidance on exercise is rarely provided under the current healthcare system [20]. Similarly, nephrologists and other healthcare providers consider exercise specialists, including physical therapists, essential for delivering exercise counseling, resources and prescriptions to patients with CKD [11, 12, 14, 21]. Thus, involvement of physical therapists in dialysis-prevention interventions is supported by both healthcare professionals and patients. The KDIGO 2024 Clinical Practice Guidelines further recommend referral to physiotherapy programs when available [22]. Participation of physical therapists in dialysis-prevention interventions has been associated with slower estimated glomerular filtration rate (eGFR) decline and a lower risk of renal replacement therapy initiation in patients with CKD [8, 23]. These findings underscore the potential importance of integrating physical therapists into dialysis-prevention care teams to optimize the delivery and benefits of exercise and physical activity programs for this population.

In this study, facilities participating in the Lifestyle-Related Disease Management Fee with physical therapists were more likely to include medical departments related to lifestyle diseases, such as nephrology and diabetology. However, for the Diabetes Dialysis Prevention Guidance and Management Fee and the CKD Dialysis Prevention Guidance and Management Fee, both of which emphasize preventing dialysis initiation, no distinctive facility characteristics were identified based on physical therapist participation. The primary reasons for non-participation in dialysis-prevention interventions were reimbursement criteria and insufficient personnel or time due to other responsibilities. As interventions were defined as those billed under the reimbursement schedule, facilities offering exercise or physical activity programs free of charge were not included. It is reasonable to infer that facilities face challenges in allocating staff or time to patients when reimbursement billing is difficult. Previous studies have also identified legislation and regulations as global barriers to implementing exercise and physical activity programs for patients with kidney disease [24]. However, less frequently cited reasons for physical therapists’ non-participation in dialysis-prevention interventions included limited interest or perceived necessity, insufficient specialized knowledge and skills, and inadequate collaboration with specialists. These findings suggest that physical therapists generally possess the motivation, knowledge, and confidence to engage in such interventions. A 2023 survey reported that 79.9% of physical therapy schools in Japan provided education on renal rehabilitation; among those not yet offering such curricula, 77.4% planned to implement them in the future [10]. This indicates that a substantial proportion of physical therapists have acquired knowledge of exercise and physical activity in patients with kidney disease. Furthermore, the annual number of published articles on exercise-based renal rehabilitation increased markedly from an average of 3 per year during 1969–1990 to 146 per year during 1991–2021 [25]. As many of these publications are likely authored by exercise professionals, including physical therapists, their knowledge and skills appear to be advancing academically. Therefore, if the healthcare system environment is appropriately structured, physical therapists are well-positioned to participate actively in dialysis-prevention interventions.

Despite the limited number of physical therapists in this study, those participating in dialysis-prevention interventions were disproportionately likely to provide aerobic exercises, resistance training, physical activity promotion, and stretching. Previous meta-analyses have shown that such interventions improve eGFR [26], and increased physical activity benefits renal outcomes [27]. Therefore, the involvement of physical therapists appears to align with current evidence. However, motivational interventions based on the Transtheoretical Model remain underutilized, despite evidence of their role in reducing CKD onset risk [28] and enhancing patient self-efficacy [29]. Broader dissemination of these behavior-change strategies may promote habitual exercise among patients with CKD.

This study has several limitations. First, the low response rate (12.9%) may limit generalizability and could have introduced selection bias. Given the voluntary nature of participation, respondents may have been more interested or engaged in promoting physical activity and exercise programs for patients with diabetes or CKD. Consequently, reasons for non-participation with relatively low frequencies—such as lack of interest or perceived need and insufficient specialized knowledge and skills—may have been underestimated, and estimates of physical therapist participation and reimbursable services may be inflated relative to the broader facility population. Moreover, the low response rate itself may indicate a generally low level of interest in this field. As information on non-responding facilities was not collected, comparisons with responding facilities and adjustment for this potential bias were not possible. Future efforts should focus on revising the healthcare system as well as increasing opportunities to disseminate dialysis-prevention information through the Japanese Society of Physical Therapy for Diabetes Mellitus, thereby enhancing the knowledge and skills of individual physical therapists. In addition, follow-up surveys are planned every few years, following increased awareness of the questionnaire’s importance, to strengthen the robustness of the results. Second, the restrictive definition of dialysis-prevention interventions may limit the findings. In this study, we defined such interventions as physical therapists’ participation in reimbursable medical services, thereby excluding facilities providing exercise or physical activity programs free of charge or voluntarily. This may underrepresent actual involvement; however, in Japan, the only reimbursable programs for exercise or activity counseling for primary conditions such as diabetes or CKD are those examined here, suggesting that the definition still captures the broader reality. Third, we did not collect geographic identifiers (e.g., region or prefecture) of responding facilities; therefore, regional representativeness, urban–rural differences, or potential clustering could not be assessed. Future surveys will include geographic information to enable such analyses. Fourth, the quality of physical therapist participation, such as the frequency, intensity, and continuity, was not evaluated, which may influence interpretation of the intervention. Further detailed investigations are warranted. Finally, the questionnaire relied on self-reported data from facility representatives, which may be subject to recall or social desirability bias.

A notable strength of this study is its nationwide assessment of physical therapists’ involvement in dialysis prevention. Previously, the extent of such participation was unclear. By documenting both the level of engagement and underlying reasons, this study addresses an important gap in the field with limited prior research. Future research will investigate the impact of physical therapists’ participation, or lack thereof, on renal outcomes.

## Conclusion

The proportion of physical therapists involved in reimbursable dialysis-prevention interventions was low, primarily due to specific billing requirements for reimbursement. Revising the current healthcare system is necessary to facilitate greater participation of physical therapists in such interventions. Future studies should investigate the impact of physical therapists’ involvement in dialysis-prevention interventions on renal outcomes.

## Supplementary Information

Below is the link to the electronic supplementary material.

Supplementary file1 (PDF 121 KB)
