# Supplementary material for: Participation of physical therapists in medical fee-based dialysis-prevention interventions: a nationwide survey in Japan
Source: Clin Exp Nephrol. 2025 Sep 19;30(1):96–108. doi: 10.1007/s10157-025-02763-z (PMC12811349; doi:10.1007/s10157-025-02763-z)
Supplement: Supplementary file 1 — Supplementary file1 (PDF 121 KB) [file 10157_2025_2763_MOESM1_ESM.pdf]

Online Resource 1 – Supplementary Table S1. Summary of the questionnaire items assessing reimbursable dialysis-prevention services, the proportion of cases involving physical therapist participation, and reasons for non-participation.

Article title: Participation of Physical Therapists in Medical Fee-Based Dialysis-Prevention Interventions: A Nationwide Survey in Japan

Journal: Clinical and Experimental Nephrology

Authors: Yuma Hirano[1–8], Kenichi Kono[2–8]*, Ren Takahashi[3–8], Yuma Tamura[4–8], Momo Takahashi[4–8], Shinsuke Imaoka[5–8], Takuo Nomura[6–8], Makoto Igaki[7–8]

Affiliations:

[1] Department of Rehabilitation Medicine, Hamamatsu University Hospital, Hamamatsu City, Shizuoka, Japan

[2] Department of Physical Therapy, International University of Health and Welfare School of Health Science at Fukuoka, Okawa City, Fukuoka, Japan

[3] Department of Rehabilitation, Kaikoukai Josai Hospital, Nagoya City, Aichi, Japan

[4] Department of Rehabilitation, Nikko Medical Center, Dokkyo Medical University, Tochigi, Japan

[5] Department of Rehabilitation, Oita Oka Hospital, Oita City, Oita, Japan

[6] Faculty of Rehabilitation, Kansai Medical University, Hirakata City, Osaka, Japan

[7] Department of Rehabilitation, Toyooka Hospital, Toyooka Public Hospitals' Association, Toyooka City, Hyogo, Japan

[8] Working Group of Research and Development, Japanese Society of Physical Therapy for Diabetes Mellitus, Minato-ku, Tokyo, Japan

Corresponding author:

Kenichi Kono

Department of Physical Therapy, International University of Health and Welfare School of Health Science at Fukuoka

137-1 Enokizu, Okawa City, Fukuoka, 831-8501, Japan

Tel: +81-92-832-1200

E-mail: kkono@iuhw.ac.jp

Supplementary material. Online survey questionnaire items

| Respondents | All participants |
|---|---|
| Q1 | Please answer one that applies to the facility where you work. |
| | 1. Hospital (affiliated with university) |

| | |
|---|---|
| | 2. Hospital (National government, public medical institutions, social insurance related organizations)<br>3. Hospital (medical corporation)<br>4. Hospital (private practice)<br>5. Clinic (medical corporation)<br>6. Clinic (private practice)<br>7. Others |
| Respondents | All participants |
| Q2 | Please one answer that applies to the number of hospital beds. |
| | 1. 600 or more beds<br>2. 300-599 beds<br>3. 100-299 beds<br>4. 1-99 beds<br>5. Without a bed |
| Respondents | All participants |
| Q3 | Please answer all clinical specialties (departments) applicable to your facility. |
| | 1. Internal medicine<br>2. Cardiology<br>3. Nephrology<br>4. Diabetes/Metabolism<br>5. Dialysis departments<br>6. Urology<br>7. Rehabilitation<br>8. Orthopedics departments<br>9. Not applicable |
| Respondents | All participants |
| Q4 | Please answer all applicable disease-specific rehabilitation fee facility criteria. |
| | 1. Cardiac Rehabilitation Fee (I)<br>2. Cardiac Rehabilitation Fee (II)<br>3. Cerebrovascular Disorders Rehabilitation Fee (I)<br>4. Cerebrovascular Disorders Rehabilitation Fee (II)<br>5. Cerebrovascular Disorders Rehabilitation Fee (III)<br>6. Disuse Syndrome Rehabilitation Fee (I)<br>7. Disuse Syndrome Rehabilitation Fee (II)<br>8. Disuse Syndrome Rehabilitation Fee (III)<br>9. Musculoskeletal Rehabilitation Fee (I) |

| | |
|---|---|
| | 10. Musculoskeletal Rehabilitation Fee (II)<br>11. Musculoskeletal Rehabilitation Fee (III)<br>12. Respiratory Rehabilitation Fee (I)<br>13. Respiratory Rehabilitation Fee (II)<br>14. Not applicable |
| Respondents | All participants |
| Q5 | Please answer how many physical therapists are employed by your organisation. |
| | 1. 30 or more<br>2. 20-29<br>3. 10-19<br>4. 1-9 |
| Respondents | All participants |
| Q6 | Please answer all disease phase of the patients covered at your facility. |
| | 1. Intensive Care Unit<br>2. Acute phase<br>3. Convalescent rehabilitation phase<br>4. Community-based integrated medical care<br>5. Community-based care<br>6. Outpatients phase<br>7. Home visits<br>8. Day-service programs<br>9. Not applicable |
| Respondents | All participants |
| Q7 | Does your institution bill for the “Lifestyle-related Disease Management”? |
| | 1. Yes, we bill for it<br>2. No, but we are preparing to bill in the future<br>3. No, and we have no plans to bill for it |
| Respondents | Participants who selected “1” in Q7 |
| Q8 | Are physical therapists involved in the team that bills for the “Lifestyle-related Disease Management”? |
| | 1. Yes, physical therapists participate<br>2. No, but we are preparing for their future participation<br>3. No, and we have no plans for their participation |
| Respondents | Participants who selected “1” in Q8 |
| Q9 | In the team that bills for the Lifestyle-related Disease Management, how, where, when, and by whom are physical therapists involved? |

| | |
|---|---|
| | (Select all that apply) |
| | 1. Providing direct exercise instruction and related interventions to patients under physician orders<br>2. Developing exercise-instruction materials used by other professionals<br>3. Training other professionals in how to deliver exercise guidance<br>4. Working in the rehabilitation room<br>5. Working outside the rehabilitation room (e.g., in the consultation room or on the ward)<br>6. Participating concurrently with disease-specific rehabilitation sessions<br>7. Participating outside of disease-specific rehabilitation hours (e.g., at lunchtime or after 5 pm)<br>8. Only a subset of highly specialized physical therapists were involved.<br>9. Many physical therapists are involved regardless of specialization<br>10. Other (please describe your approach in detail) |
| Respondents | Participants who selected “1” in Q8 |
| Q10 | Please answer all applicable questions. What is the nature of the exercise instruction that the physical therapist will be involved in? |
| | 1. Aerobic exercise and exercise prescription<br>2. Resistance exercise and exercise prescription<br>3. Guidance on increasing physical activity（including the use of activity monitors such as accelerometers and pedometers）<br>4. Stretching exercises<br>5. Motivational counseling based on the stages of behavior change<br>6. Other (please describe your approach in detail) |
| Respondents | Participants who selected “1” in Q8 |
| Q11 | Of all patients billed under the Lifestyle-related Disease Management, approximately what percentage receive involvement from physical therapists? |
| | 1. ≥ 75 %<br>2. ≥ 50 % but < 75 %<br>3. ≥ 25 % but < 50 %<br>4. < 25 % |
| Respondents | Participants who selected “2” or “3” in Q8 |
| Q12 | Please answer why physical therapists are not involved in the calculation of Lifestyle -related Disease Management. |
| | 1. The assignment of physical therapists is not a requirement for meeting the reimbursement criteria |

| | |
|---|---|
| | 2. They were not assigned a role by the dialysis-prevention team<br>3. There is insufficient personnel or time available<br>4. Low cost-effectiveness<br>5. It does not directly generate revenue for the rehabilitation department<br>6. Lack of collaboration with the department physicians (internal medicine specialists)<br>7. No physical therapists with the necessary specialized knowledge and skills are available<br>8. Lack of perceived interest or necessity within the physical therapy department.<br>9. Other (please specify)<br>10. Unknown |
| Respondents | Participants who selected "2" or "3" in Q7 |
| Q13 | Please answer the reason why the Lifestyle-related Disease Management is not calculated at your medical institution. |
| | 1. Failure to meet the facility criteria (e.g., ≥ 200 licensed beds)<br>2. No relevant department or specialist physicians available<br>3. Insufficient non-physician staff and inability to coordinate multidisciplinary collaboration<br>4. No eligible patients<br>5. Unaware of the existence of the Lifestyle-related Disease Management Fee<br>6. Other (please specify)<br>7. Unknown |
| Respondents | All participants |
| Q14 | Does your institution bill for the "Diabetes Dialysis Prevention Guidance and Management"? |
| | 1. Yes, we bill for it<br>2. No, but we are preparing to bill in the future<br>3. No, and we have no plans to bill for it |
| Respondents | Participants who selected "1" in Q14 |
| Q15 | Are physical therapists involved in the dialysis-prevention clinical team that bills for the Diabetes Dialysis Prevention Guidance and Management ? |
| | 1. Yes, physical therapists participate<br>2. No, but we are preparing for their future participation<br>3. No, and we have no plans for their participation |
| Respondents | Participants who selected "1" in Q15 |

| | |
|---|---|
| Q16 | In the team that bills for the Diabetes Dialysis Prevention Guidance and Management, how, where, when, and by whom are physical therapists involved? |
| | 1. Providing direct exercise instruction and related interventions to patients under physician orders<br>2. Developing exercise-instruction materials used by other professionals<br>3. Training other professionals in how to deliver exercise guidance<br>4. Working in the rehabilitation room<br>5. Working outside the rehabilitation room (e.g., in the consultation room or on the ward)<br>6. Participating concurrently with disease-specific rehabilitation sessions<br>7. Participating outside of disease-specific rehabilitation hours (e.g., at lunchtime or after 5 pm)<br>8. Only a subset of highly specialized physical therapists were involved.<br>9. Many physical therapists are involved regardless of specialization<br>10. Other (please describe your approach in detail) |
| Respondents | Participants who selected “1” in Q15 |
| Q17 | Please answer all applicable questions. What is the nature of the exercise instruction that the physical therapist will be involved in? |
| | 1. Aerobic exercise and exercise prescription<br>2. Resistance exercise and exercise prescription<br>3. Guidance on increasing physical activity（including the use of activity monitors such as accelerometers and pedometers）<br>4. Stretching exercises<br>5. Motivational counseling based on the stages of behavior change<br>6. Other (please describe your approach in detail) |
| Respondents | Participants who selected “1” in Q15 |
| Q18 | Of all patients billed under the Diabetes Dialysis Prevention Guidance and Management, approximately what percentage receive involvement from physical therapists? |
| | 1. $\geq$ 75 %<br>2. $\geq$ 50 % but < 75 %<br>3. $\geq$ 25 % but < 50 %<br>4. < 25 %<br>5. Only when billing the “Guidance of Patients with Severe Renal Impairment (eGFR < 45 mL/min/1.73 m²)” |
| Respondents | Participants who selected “1” in Q15 |

| | |
|---|---|
| Q19 | Of the Diabetes Dialysis Prevention Guidance and Management, has the Guidance of Patients with Severe Renal Impairment been calculated? |
| | 1. Yes<br>2. No |
| Respondents | Participants who selected “1” in Q19 |
| Q20 | Approximately how many patients per month are billed under the Guidance of Patients with Severe Renal Impairment? |
| | 1. 20 or more<br>2. 15–19<br>3. 10–14<br>4. 5–9<br>5. Fewer than 5 |
| Respondents | Participants who selected “2” or “3” in Q15 |
| Q21 | Why are physical therapists not involved in billing for the Diabetes Dialysis Prevention Guidance and Management or the Guidance of Patients with Severe Renal Impairment at your institution? |
| | 1. The assignment of physical therapist is not a requirement for meeting the reimbursement criteria<br>2. They were not assigned a role by the dialysis-prevention team<br>3. It is difficult to provide instruction on the same day as other healthcare professionals.<br>4. There is insufficient personnel or time available.<br>5. Low cost-effectiveness<br>6. It does not directly generate revenue for the rehabilitation department<br>7. Lack of collaboration with the department physicians (internal medicine specialists)<br>8. No physical therapists with the necessary specialized knowledge and skills are available<br>9. Lack of perceived interest or necessity within the physical therapy department.<br>10. Other (please specify)<br>11. Unknown |
| Respondents | Participants who selected “2” or “3” in Q14 |
| Q22 | Why does your institution not bill for the Diabetes Dialysis Prevention Guidance and Management? |
| | 1. Failure to meet the facility criteria<br>2. No dedicated physician (department or specialist) available |

| | |
|---|---|
| | 3. Unable to establish a dialysis-prevention clinical team<br>4. No eligible patients<br>5. Unaware of the existence of the Diabetes Dialysis Prevention Guidance and Management Fee<br>6. Do not perceive a need to bill for it as a medical institution<br>7. Other (please specify)<br>8. Unknown |
| Respondents | All participants |
| Q23 | Does your institution bill for the “CKD Dialysis Prevention Guidance and Management”? |
| | 1. Yes, we bill for it<br>2. No, but we are preparing to bill in the future<br>3. No, and we have no plans to bill for it |
| Respondents | Participants who selected “1” in Q23 |
| Q24 | Are physical therapists involved in the dialysis-prevention clinical team that bills for the CKD Dialysis Prevention Guidance and Management? |
| | 1. Yes, physical therapists participate<br>2. No, but we are preparing for their future participation<br>3. No, and we have no plans for their participation |
| Respondents | Participants who selected “1” in Q24 |
| Q25 | In the team that bills for the CKD Dialysis Prevention Guidance and Management, how, where, when, and by whom are physical therapists involved? |
| | 1. Providing direct exercise instruction and related interventions to patients under physician orders<br>2. Developing exercise-instruction materials used by other professionals<br>3. Training other professionals in how to deliver exercise guidance<br>4. Working in the rehabilitation room<br>5. Working outside the rehabilitation room (e.g., in the consultation room or on the ward)<br>6. Participating concurrently with disease-specific rehabilitation sessions<br>7. Participating outside of disease-specific rehabilitation hours (e.g., at lunchtime or after 5 pm)<br>8. Only a subset of highly specialized physical therapists were involved.<br>9. Many physical therapists are involved regardless of specialization<br>10. Other (please describe your approach in detail) |
| Respondents | Participants who selected “1” in Q24 |

| | |
|---|---|
| Q26 | Please answer all applicable questions. What is the nature of the exercise instruction that the physical therapist will be involved in? |
| | 1. Aerobic exercise and exercise prescription<br>2. Resistance exercise and exercise prescription<br>3. Guidance on increasing physical activity（including the use of activity monitors such as accelerometers and pedometers）<br>4. Stretching exercises<br>5. Motivational counseling based on the stages of behavior change<br>6. その他（ ） |
| Respondents | Participants who selected “1” in Q24 |
| Q27 | Of all patients billed under the CKD Dialysis Prevention Guidance and Management, approximately what percentage receive involvement from physical therapists? |
| | 1. ≥ 75 %<br>2. ≥ 50 % but < 75 %<br>3. ≥ 25 % but < 50 %<br>4. < 25 % |
| Respondents | Participants who selected “1” in Q24 |
| Q28 | Approximately how many patients per month are billed under the CKD Dialysis Prevention Guidance and Management? |
| | 1. 20 or more<br>2. 15–19<br>3. 10–14<br>4. 5–9<br>5. Fewer than 5 |
| Respondents | Participants who selected “2” or “3” in Q24 |
| Q29 | Why are physical therapists not involved in billing for the CKD Dialysis Prevention Guidance and Management at your institution? |
| | 1. The assignment of physical therapists is not a requirement for meeting the reimbursement criteria<br>2. They were not assigned a role by the dialysis-prevention team<br>3. It is difficult to provide instruction on the same day as other healthcare professionals.<br>4. There is insufficient personnel or time available.<br>5. Low cost-effectiveness<br>6. It does not directly generate revenue for the rehabilitation department |

| | |
|---|---|
| | 7. Lack of collaboration with the department physicians (internal medicine specialists)<br>8. No physical therapists with the necessary specialized knowledge and skills are available<br>9. Lack of perceived interest or necessity within the physical therapy department.<br>10. Other (please specify)<br>11. Unknown |
| Respondents | Participants who selected “2” or “3” in Q23 |
| Q30 | Why does your institution not bill for the CKD Dialysis Prevention Guidance and Management? |
| | 1. Failure to meet the facility criteria<br>2. No dedicated physician (department or specialist) available<br>3. Unable to form the dialysis-prevention clinical team (required physicians with specified years of experience, nurses/public health nurses, and registered dietitians)<br>4. Dialysis-prevention team members have not completed the required training for preventive guidance<br>5. No eligible patients<br>6. Unaware of the existence of the CKD Dialysis Prevention Guidance and Management Fee<br>7. Do not perceive a need to bill for it as a medical institution<br>8. Other (please specify)<br>9. Unknown |

*CKD* chronic kidney disease
